# Paroxysmal extreme pain disorder associated with a mutation in SCN9A gene – Case report and own experiences

**DOI:** 10.3389/fneur.2024.1477982

**Published:** 2024-12-06

**Authors:** Mateusz Szczupak, Jolanta Wierzchowska, Maria Cimoszko-Zauliczna, Jacek Kobak, Justyna Kosydar-Bochenek, Wojciech Radys, Agnieszka Szlagatys-Sidorkiewicz, Dorota Religa, Sabina Krupa-Nurcek

**Affiliations:** ^1^Department of Anesthesiology and Intensive Care, Copernicus Hospital in Gdańsk, Gdańsk, Poland; ^2^Department of Otolaryngology, Faculty of Medicine, Medical University of Gdańsk, Gdańsk, Poland; ^3^Institute of Health Sciences, College of Medical Sciences of the University of Rzeszów, Rzeszów, Poland; ^4^Department of Gastroenterology, Allergology and Child Nutrition, Medical University of Gdańsk, Gdańsk, Poland; ^5^Department of Neurobiology, Care Science and Society (NVS), I Karolinska Institute, Stockholm, Sweden; ^6^Department of Surgery, Institute of Medical Sciences, Medical College of Rzeszów University, Rzeszów, Poland

**Keywords:** pain, paroxysmal extreme pain syndrome, SCN9A gene, genetic variability, chronic pain

## Abstract

**Introduction:**

Pain is an unpleasant sensory and emotional experience, influenced by various factors. Paroxysmal extreme pain disorder (PEPD) is a rare genetic condition characterized by sudden bouts of pain accompanied by autonomic symptoms.

**Material, methods and aim:**

This manuscript presents the case of a 9-year-old boy with paroxysmal extreme pain syndrome and provides a review of the literature. Additionally, a genealogical analysis of the boy’s family was conducted to determine the total number of affected family members. The clinical data included an analysis of genetic tests to identify the mutation confirming PEPD.

**Result and conclusion:**

A mutation in the SCN9A gene causes the disease, and due to the small number of patients worldwide (around 500, according to literature reports), an effective method of preventing extreme pain attacks had not been established at the time of writing this manuscript. Based on information from scientific sources and the authors’ experiences, it can be firmly stated that various, often difficult-to-identify factors cause paroxysmal extreme pain. This syndrome necessitates further research and the exploration of effective treatment methods.

## 1 Introduction

Pain is an unpleasant sensory and emotional experience associated with or resembling the sensation linked to current or potential tissue damage (1). It is a personal experience influenced by biological, social, and psychological factors to varying degrees (1). Paroxysmal extreme pain disorder (PEPD) is a rare genetic condition characterized by paroxysmal, burning pain in the rectal, ocular, and mandibular regions, accompanied by symptoms of the autonomic nervous system such as skin flushing and bradycardia (2, 3).

Paroxysmal extreme pain was first described in the literature by Hayden et al. in 1959 as a familial disorder characterized by episodes of very brief excruciating pain localized to the rectal region. The pain attacks were accompanied by redness of the buttocks and lower extremities, pain in the eyeballs and submaxillary region, and redness of the eyelids and skin around the eyes (4).

The autonomic symptoms of paroxysmal extreme pain syndrome often manifest as redness on the skin in a harlequin character and can be present from early infancy. Individuals with this syndrome may experience episodes of fainting accompanied by a slow heart rate, temporary cessation of breathing, and sudden cardiac arrest. In addition, symptoms of the syndrome may include tonic non-epileptic seizures. Excruciating, piercing, or burning pain in the rectum, jaw, and eye area may develop later in patients’ lives. Triggers for pain attacks can include defecation, urination, exposure to cold wind, eating a meal, experiencing emotions, undergoing a rectal or gynecological examination, or even physical touch. The pain can range from a few seconds to several hours. Fertleman et al. suggest that these symptoms can be relieved entirely with carbamazepine (5). The estimated incidence of PEPD is currently less than 1 in 1,000,000 cases (6).

**Figure 1:**
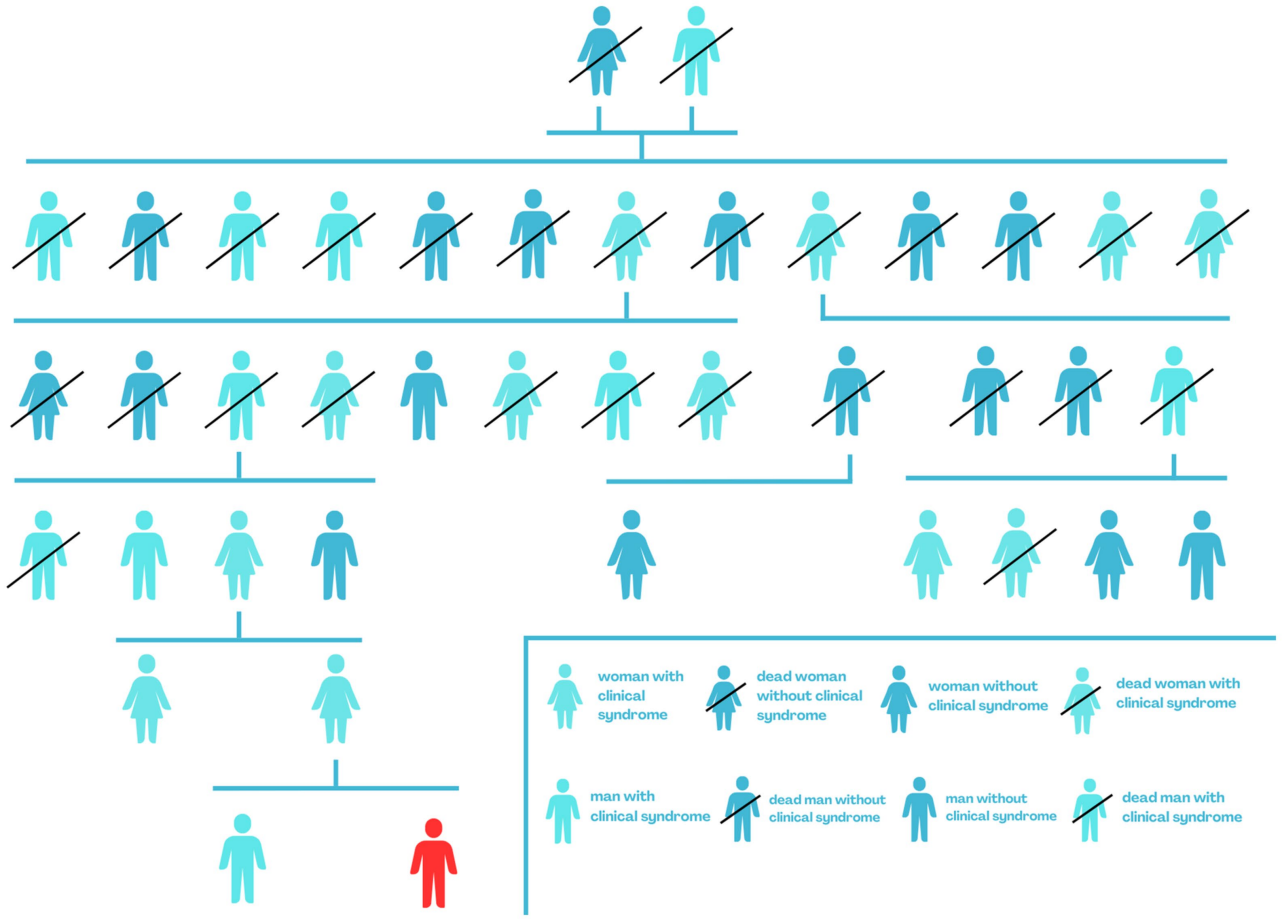
Genealogical tree of the boy’s family - red indicates the PEPD case described.

During the case presentation for a 9-year-old boy in this manuscript, we collected clinical data and took a detailed history from immediate family members. We included the age at which the first PEPD symptoms occurred, the symptoms accompanying, the results of genetic testing, and the treatment used. Additionally, we presented a family tree of the boy’s family, broken down by gender and labeling members burdened with PEPD (Figure 1).

## 2 Aim of the study

The aim of the study was presentation a case of a 9-year-old boy admitted to the Department of Gastroenterology, Allergology, and Child Nutrition of the Medical University of Gdansk due to complications with constipation, fecal stones, and excessive pathological rectal dilatation. These symptoms were a result of paroxysmal extreme pain syndrome and were linked to psychological fears of experiencing pain after defecation. The authors reviewed existing literature to determine the management and treatment of a patient with this syndrome.

## 3 Materials and methods

To write this manuscript, we conducted a review of articles available in PubMed, Google Scholar, and the Mendeley search engine. We used keywords such as paroxysmal extreme pain syndrome, SCN9A gene mutation, and genetically determined pain. Out of 30 review articles and articles presenting PEPD cases that we found and analyzed, we selected those that, in the authors’ opinion, were related to the topic of this manuscript and were valuable sources of information. The discussion excluded conditions with similar pathophysiology to paroxysmal extreme pain syndrome, as well as those related to genetic factors not directly associated with PEPD. The article referenced 17 publications and scientific reports. Additionally, it presented a case of a 9-year-old patient hospitalized at the Department of Gastroenterology, Allergology, and Child Nutrition of the Medical University of Gdansk due to symptoms and complications of paroxysmal extreme pain syndrome.

## 4 Pathophysiology of the paroxysmal extreme pain syndrome

Paroxysmal extreme pain syndrome (PEPD) is a rare, genetically determined, and autosomal dominantly inherited pain disorder associated with a mutation in the SCN9A gene. This gene encodes the voltage-gated sodium channel Nav1.7. Disturbances in normal pain sensation occur due to changes in the properties of voltage-gated sodium channels, which leads to impaired inactivation of the sodium channel (2, 6–8). The Nav1.7 channel is highly expressed in peripheral somatic and visceral sensory neurons, dorsal root ganglion (DRG) nociceptive neurons, trigeminal ganglia, olfactory sensory neurons, and sympathetic ganglia (7, 8). The SCN9A gene has different mutations that are associated with various symptoms. These symptoms can range from congenital insensitivity to pain, primary erythromelalgia, and febrile convulsions to small fiber sensory neuropathy or paroxysmal extreme pain syndrome. These mutations result in a wide range of symptoms in different disorders (9, 10).

## 5 Case presentation

**Figure 2:**
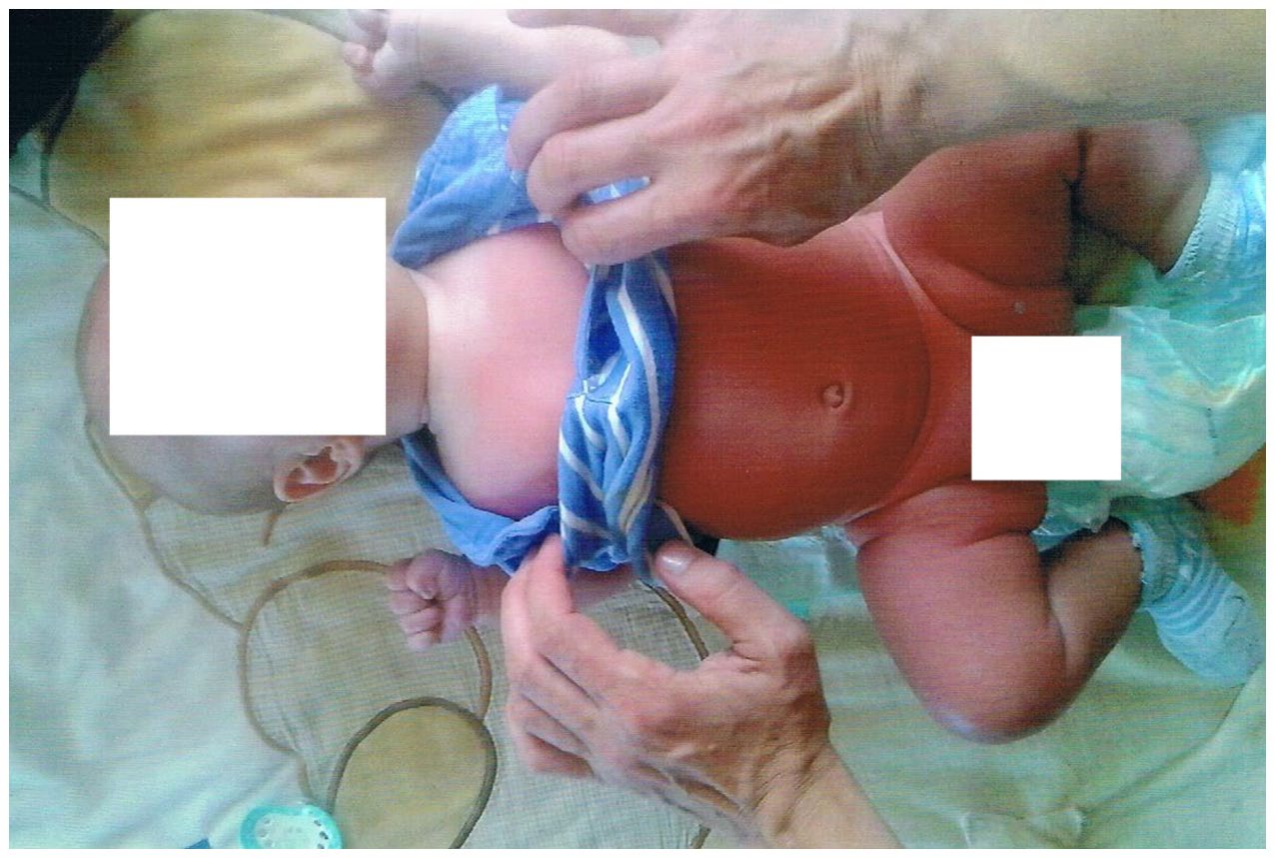
The child face was veiled for anonymity.

**Figure 3:**
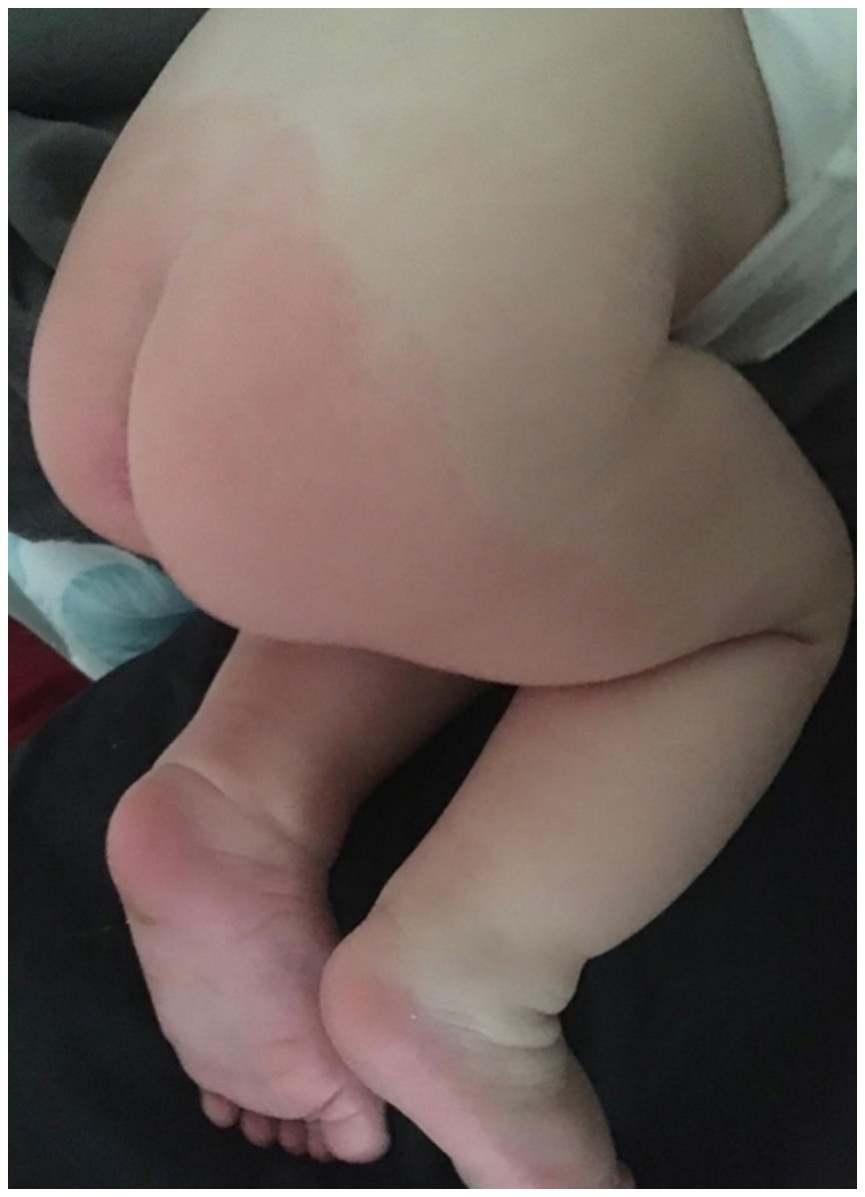
Redness of the skin on the buttocks and lower limbs after defecation.

A 9-year-old boy was admitted to the Department of Gastroenterology, Allergology, and Pediatric Nutrition at the Medical University of Gdansk with paroxysmal extreme pain syndrome diagnosed and confirmed by genetic testing. The baby was delivered by cesarean section at 35 weeks’ gestation, weighing 2,540 grams. The Apgar scores were 5 at 1 min, 8 at 5 min, and 9 at 10 min after birth. Following birth, the boy experienced respiratory issues in the form of apnea, leading to a diagnosis of respiratory distress syndrome. The child needed non-invasive ventilation (NIV) first, followed by passive oxygen therapy using a face mask. During the diagnosis of the causes of respiratory distress, it was confirmed that there was no intrauterine infection. The boy was given nutrition through a vein until the 7th day of life. In addition to the initial clinical signs in the neonatal period, the baby exhibited decreased muscle tone and low activity. As a result, the doctors decided to expand the diagnosis by examining the cerebrospinal fluid, which showed no abnormalities. A heart echo performed in the first month of the baby’s life revealed no heart defects or cardiomyopathy. At 2.5 months old, an MRI of the head was conducted, and no pathological changes in the brain structures were found. The patient was admitted to the Department of Gastroenterology, Allergology, and Child Nutrition due to fecaliths causing persistent constipation. The boy first showed symptoms of PEPD at 2.5 months old, which were related to touch (Figure 2). The boy experienced episodes of sudden cardiac arrest with successful resuscitation, displaying symptoms such as reddening of the skin, intense crying, and repeated respiratory arrest. During the investigation following the cardiac arrest, the team of doctors was unable to determine whether the direct cause was the Nav 1.7 mutation or if it resulted from acute pain, which could have been a contributing factor. These episodes occurred three times during his first year of life. At 1.5 years old, the boy started experiencing headaches triggered by crying or hitting the eyeball. As he grew older, he developed skin lesions, characterized by redness in the buttocks and lower extremities, which were caused by defecation (Figure 3). The family mentioned that the first extreme pain episode may have occurred at birth, leading to apneic episodes shortly after birth.

**Figure 4:**
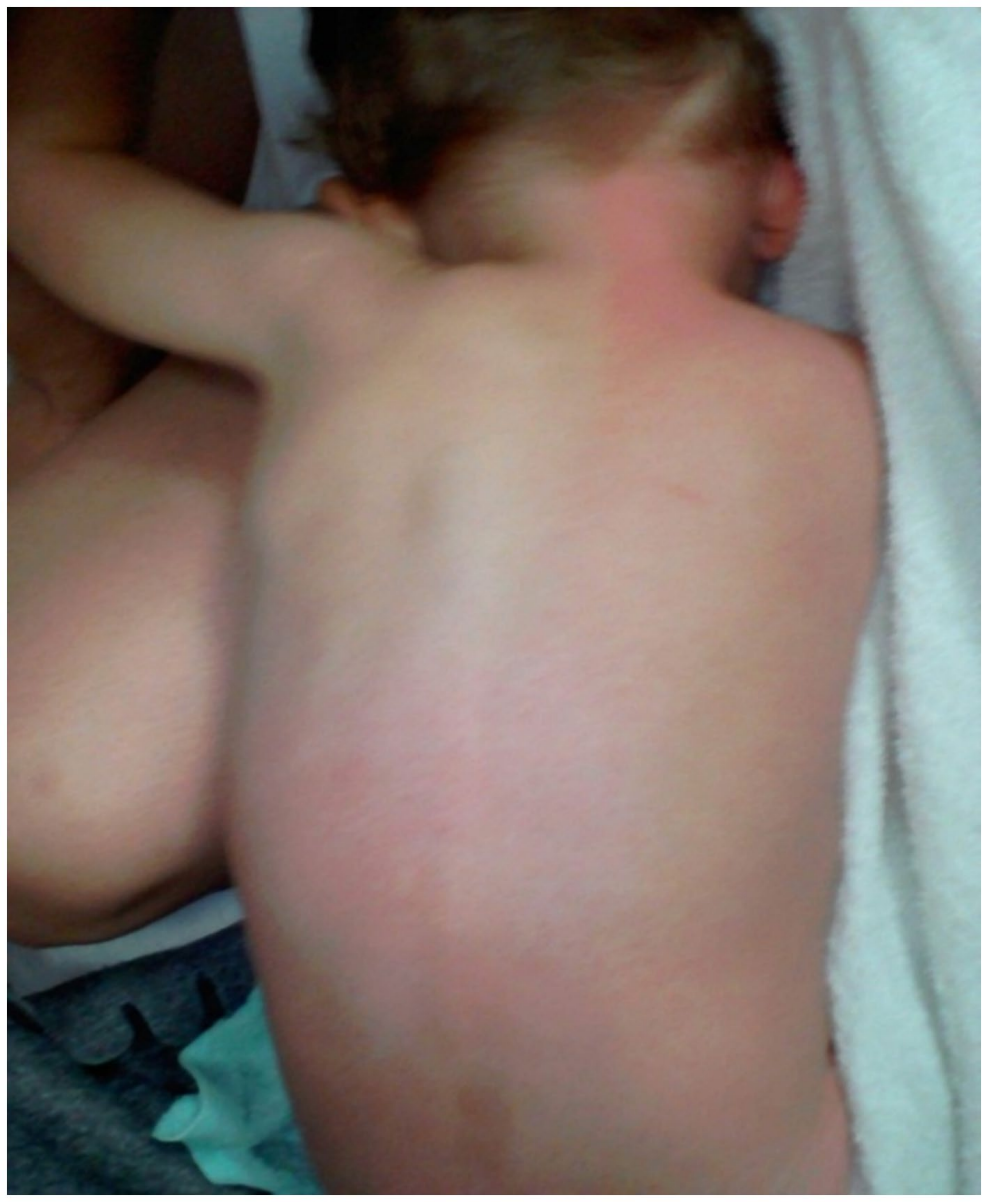
Reddening of the skin on the trunk due to a mechanical stimulus.

At the age of 9, the boy experiences intense and localized pain in the anal area and other regions when passing stool. This pain is accompanied by redness on the trunk, making daily activities challenging (Figure 4). He has been diagnosed with constipation after undergoing various tests at the Department of Gastroenterology, Allergology, and Child Nutrition. The treatment involved a manual stool removal procedure under general anesthesia, along with macrogol therapy, which has shown positive results.

In the case of a 9-year-old boy, genetic testing at the Medizinisch Genetische Zentrum in Munich revealed a c.3892G > T mutation (p.Val1298Phe) in the SCN9A gene, confirming the original clinical diagnosis. The boy has been treated with non-steroidal anti-inflammatory drugs (ibuprofen) and paracetamol, but these did not have a satisfactory therapeutic effect. The family was advised to start treatment with carbamazepine as per current recommendations, and to continue using macrogol and maintaining a fiber-rich diet. We present the child’s face with the consent of his parents in order to illustrate the level of pain the boy is experiencing. The age of the baby at the photo is about 9 months (Figure 2).

Despite the doctor’s suggestions and recommendations for treatment to improve the boy’s clinical condition, the parents, who are the legal guardians, did not agree to start treatment with carbamazepine. Their decision was influenced by the lack of positive results from using carbamazepine to control pain in other family members who also suffer from paroxysmal extreme pain syndrome.

## 6 Discussion

Paroxysmal extreme pain syndrome is a rare, genetically determined disorder caused by a sodium channelopathy. It has been documented in only 500 individuals in the literature. This channelopathy leads to both intense pain and small nerve fiber neuropathy, or hereditary erythromelalgia (11).

The diagnosis of paroxysmal extreme pain syndrome is based on characteristic symptoms, the progression of the condition, and genetic testing. Establishing the diagnosis in the early stages can be challenging and may require various diagnostic procedures, including laboratory tests, imaging, and genetic testing. The initial symptoms may manifest shortly after birth, during the neonatal period. Individuals affected by paroxysmal extreme pain syndrome have the same intellectual and cognitive capabilities as the general population (6, 12). Based on available information and literature reports, 11 genetic mutations have been identified as the cause of PEPD (13).

The following table (Table 1) summarizes several cases of paroxysmal extreme pain syndrome described in the literature. It includes demographic information, the specific variant of the SCN9A gene mutation, and the symptoms characteristic of each patient with PEPD. Additionally, the table outlines the treatment options used for pain attacks and their effectiveness.

**Table 1:** Cases of paroxysmal extreme pain were selected and described in the literature, including demographic information, SCN9A gene mutation variants, characteristic symptoms, and the treatments used.

Age	Sex	Nationality	Gene	Variant/Nucleotide	Type of mutation	Symptoms	Factor causing of symptoms	Treatment	Result	Reference
10 months	Girl	Slovakia	SCN9A	c.4382 T > A (p.Ile1461Asn)	Heterozygous, *de novo*	Pain, transineto loss of consciousness, cynosic, epilepsy, 6-s sinus pause in ECG holter, short syncope, erythema	Defecation, rectal tube insertion, small injuries	Pacemaker implantation, carbamazepine	Reduce the frequency of symptoms	(16)
2 years and 6 months	Girl	China	SCN9A	c.4384 T > A (p.F1462l)	Heterozygous, *de novo*	Seizures, flushing and sweating of the skin on one side face and trunk, paroxysmal headache, cyanosis if the face and lips, constipation	Stimulation of the perineum, defecation, touching the body	Carbamazepine	Reduce the frequency of symptoms	(3)
3 years	Girl	Slovenia	SCN9A	c.554G > A (p.Arg185His)	Heterozygous, inherited paternally	Very painful voiding without any other autonomic manifestations, sweating	Urination	Carbamazepine	Reduce the frequency of symptoms. After a year of therapy the pain disappeared	(10)*
5 years	Girl	Japan	SCN9A	c.5218G > C (p.Val1740Leu)	Heterozygous, inherited	Unilateral, mainly left-sided forehead, temple and retro-orbital region, lacrimation, nasal congestion, rhinorrhea, horsehead and facial flushing, agitation	Hitting of the head or body, bath, change of temperature, sleep	Carbamazepine	Reduce the frequency of symptoms. After 2 weeks of treatment the symptoms disappeared	(17)
44 years	Woman	Poland	SCN9A	c.3892G > T (p.Val1298Phe)	Heterozygous, inherited, autosomal dominant	Pain of the tearing character located in different parts of the body including perineum area, shortness of breath, flushing of half or part of the body, hot feeling on the side of flushing, headache, lacrimation	Defecation, pressure, scratch, stressful situation	Carbamazepine Topiramate and pregabalin	Lack of therapeutic effect. The symptoms have improved partially	(11)**
61 years	Woman	United States, Puerto Rico ancestry	SCN9A	c.2159 T > A (p.I720K)	Heterozygous Inherited	Acute facial pain, chronic pain, fibromyalgia, constipation, headache, facial swelling, eye weakness, forehead paralysis, eye pain, ptosis, rectal pain, syncope	Smiling, brushing teeth, chewing, cold, defecation	Oxcarbazepine Gabapentin Lacosamide Nontraditional therapies	Stevens-Johnson syndrome side effect Lack of effect, only sedating effect Patient’s lack of consent to treatment Effective and treating the pain on this case	(15)***

*This case of PEPD is noteworthy due to small-fiber neuropathy, which is likely associated with Nav 1.7 despite being an atypical lesion for PEPD. The Trigger (voiding) is uncommon, and the characteristic skin flushing is lacking. Finally, pain disappears after one year of treatment with carbamazepine, which is unusual. **This patient is a relative (grandmother) of the case presented by the authors. ***This is an unusual case due to the age at which symptoms first appeared. The authors of the manuscript argue that it is inappropriate to classify it as paroxysmal extreme pain syndrome (PEPD). Although the original article referred to this condition as PEPD, this diagnosis seems questionable.

As of the date of writing this manuscript, the authors have not found an effective treatment for paroxysmal extreme pain syndrome in the scientific reports and literature they reviewed. However, available information sources recommend the use of sodium channel inhibitors, such as antiepileptic drugs, tricyclic antidepressants, and serotonin and norepinephrine reuptake inhibitors in the treatment of PEPD. Carbamazepine is the preferred drug for reducing the frequency and number of PEPD seizures and, in some cases, eliminating them (6, 11). In a study titled “The Role of Voltage-Gated Sodium Channel in Pain Signaling,” Bennett et al. confirm the theory of carbamazepine’s effectiveness in reducing the frequency and severity of pain attacks in PEPD. According to the authors, carbamazepine is an effective treatment for reducing the frequency and severity of pain attacks in PEPD. It has also been shown to reduce persistent sodium current generated by PEPD-associated mutant Na_v_1.7. This is consistent with the effect of carbamazepine in stabilizing the inactive state of VGSCs in a use-dependent manner, which can reduce repetitive firing (8). Drugs like gabapentin, topiramate, and lamotrigine have shown partial efficacy in some cases. Amitriptyline, clonidine, and intravenous lidocaine injections have not had the desired effect. In children experiencing severe seizures, improvement can be achieved by using a 1:1 mixture of nitrous oxide and oxygen (6, 14). Finally, pelvic floor muscle exercises and constipation prevention have been reported to control pain attacks effectively (15), according to Cannon et al. The available literature is not extensive when it comes to the topic of extreme pain because this phenomenon is extremely rare in the world.

## 7 Conclusion

Extreme pain resulting from a rare genetic condition linked to a sodium channel gene mutation presents a significant challenge for medical professionals. Despite various treatment approaches, including a wide array of medications, complete relief for patients experiencing extreme pain attacks has not been found. This underscores the need for additional research into this syndrome and the quest for an effective drug to manage the condition.

According to the authors, managing a patient with a rare condition should involve effective and open communication between the patient, their caregivers, and the treatment team. Proper implementation of medical recommendations is crucial for mitigating the factors that trigger the activation of pathological sodium channels, offering a chance to prevent further episodes of extreme pain syndrome.

## Data Availability

The raw data supporting the conclusions of this article will be made available by the authors, without undue reservation.
